# Correlations between Health Insurance Status and Risk Factors for Cardiovascular Disease in the Elderly Asian American Population

**DOI:** 10.7759/cureus.2303

**Published:** 2018-03-10

**Authors:** Iris Cheng, Wesley E Ho, Benjamin K Woo, John T Tsiang

**Affiliations:** 1 Community Programs Office, UCLA, Los Angeles, CA; 2 UCLA, Los Angeles, Ca, UCLA, Los Angeles, CA; 3 School of Medicine, Case Western Reserve University

**Keywords:** elderly, insurance, cardiovascular disease, asian american

## Abstract

Background

Asian Americans are often seen as a model minority; however, the group faces significant cultural, language, and financial barriers to adequate health care access. Assessing health insurance’s impact on cardiovascular disease risk factors among older Asian Americans may provide support for further research and intervention development focused on improving insurance enrollment. The authors sought to examine the associations between elevated blood pressure and body mass index and insurance coverage.

Methods

Individuals attended health fairs held by a student-led organization in Southern California between 2008 and 2011. Age and insurance status were obtained through participant questionnaires. Participants’ blood pressure and body mass index were measured. Analyses identified health and insurance associations.

Results

In total, 53.8% of respondents were 60 years or older. Of these, 30.9% had an elevated blood pressure and 36.6% had an elevated body mass index. Of respondents 60 years or older, 52.0% had health insurance. Both elevated blood pressure (p = 0.04) and body mass index (p = 0.03) were significantly associated with lacking insurance.

Conclusions

Insured participants were less likely to have elevated blood pressure and body mass index measurements, supporting a positive correlation between having insurance and less risk factors for cardiovascular disease. These findings provide incentives for further research into the importance of health insurance in preventative health care.

## Introduction

Access to healthcare is a contentious issue in the current sociopolitical landscape in America, as the health care repeal and reform paradigm may result in millions of Americans becoming uninsured [1]. Studies have already determined the negative consequences associated with inadequate or insufficient health care access for health in certain subpopulations [2]. Diseases that are prevalent include hypertension and obesity. These chronic health conditions are positively correlated with many other diseases in older populations, such as cardiovascular disease (CVD) [3] and type II diabetes mellitus [4].

Asian Americans are commonly seen as a model minority [5], with fewer socioeconomic and health problems compared to the average minority. However, Asian Americans face many barriers to health care access, including language, cultural, insurance, and financial barriers [5]. With regards to health issues, cardiovascular disease (CVD) was the second leading cause of death among Asian Americans, after malignant neoplasms. CVD still accounts for 23.5% of all Asian American deaths in the United States between 2003 and 2011 [6]. One way to reduce CVD mortality is to understand how different factors, especially insurance status, can reduce the rate of these risk factors among Asian Americans.

The authors are unaware of any literature that links health insurance to health problems in Asian American populations. In this study, data obtained from Asian Americans living in the Greater Los Angeles area was used to determine correlations between health insurance status and overall health, as measured by blood pressure (BP) and body mass index (BMI).

## Materials and methods

In this study, 1,605 Asian adult inhabitants of the Greater Los Angeles area were recruited to participate in health fairs organized by Asian Pacific Health Corps (APHC) from 2008 to 2011. The health fairs were advertised by distributing flyers at local supermarkets and through advertisements in local newspapers. The flyers were targeted to recruit Asian American participants, but services were not limited solely to people of Asian descent.

At the health fairs, participants completed language-appropriate socioeconomic and demographic questionnaires. Trained student volunteers using portable scales and stadiometers measured the heights and weights of the participants. BMI and body fat percentage were calculated (kg/m2) using an Omron HBF-306-E Body Fat Analyzer (Omron, Kyoto, Japan). Participants were then classified as normal, overweight, or obese [7]. Student volunteers certified by the American Heart Association (AHA) also measured the blood pressure of participants in a seated position on the arm of the participant’s choice using the appropriately sized cuff and the bell of a 3M Littmann stethoscope (3M, Minnesota, US). The student volunteer then assessed whether the participants were normal, pre-hypertensive, or hypertensive in accordance with AHA guidelines [8]. Participants with elevated BP, BMI, and/or body fat were then queried as to whether they regularly saw a physician. Those who did not were encouraged to make an appointment and were provided with a list of free clinics in the surrounding area.

This was an institution review board-exempt cross-sectional study. The data was collected in a de-identified manner with consent from the participants and no interventions were performed on the study population. Descriptive statistics were used to analyze the demographics of the population, and chi-square analysis was performed to assess whether insurance status was significantly associated with increased prevalence of hypertension and obesity. Since the prevalence of hypertension and other cardiovascular risk factors increases with age [9], adults younger than 60 years old were excluded in order to establish the effect of health insurance on vulnerable segments of the population.

## Results

A total of 1,605 Asian Americans had their BP and BMI measured at an APHC health fair. More than half (977) of the participants were 60 years of age or older. Health insurance status was self-reported by 864 participants. Of those, 449 participants had health insurance, and 415 participants did not.

**Figure 1:**
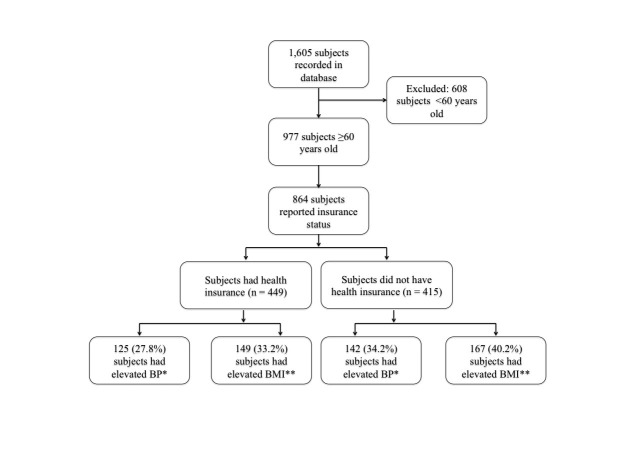
Flow diagram depicting study population, Southern California, 2008-2011 *p = 0.0427, **p = 0.0314. BP = blood pressure, BMI = body mass index.

Within the population of participants over the age of 60 years and having health insurance, 125 (27.8%) had elevated BP and 149 (33.2%) had elevated BMI. In contrast, of those over the age of 60 and without health insurance, 142 (34.2%) had elevated BP and 167 (40.2%) had elevated BMI. Analysis reveals that the lack of health insurance was significantly associated with elevated BP (*p* = 0.0427). Lack of health insurance and elevated BMI were also significantly associated (*p *= 0.0314) (Figure 1).

## Discussion

Lack of health insurance is significantly associated with elevated BP and BMI in the elderly Asian American population. The authors used these two values as proxy measurements for the overall health status of patients seen due to their recognition as risk factors for CVD. Our results thus suggest that health insurance is protective against chronic health conditions in general. The authors propose that higher rates of health insurance coverage are necessary for improving the overall health of the Asian American population.

There are several postulated reasons for why Asian Americans and other ethnic minorities tend to lack health insurance. One problem is the language barrier. The current insurance market is confusing for non-native English speakers to navigate [10]. Compared to other ethnic minorities, Asian Americans are more likely to be self-employed [11], and thus less likely to receive health insurance through their workplace. Lastly, while Asian Americans as a racial category are seen as a model minority, the heterogeneity of the group can lead to false generalizations that may not apply even within ethnic groups [12]. Thus, financial difficulties may be a barrier to health insurance, even when the group appears to be socioeconomically successful.

A caveat of this study is its limited external validity. Only Asian Americans were surveyed; respondents were primarily ethnically Chinese Americans. Only one demographic factor was surveyed in this study; it is unknown whether other factors such as socioeconomic status, sex, or ethnicity may ultimately affect the results. Furthermore, the study is limited to a geographic area with an established, thriving community of Asian Americans. It is unknown whether a more isolated population will have stronger or weaker associations between health insurance and health status. Additionally, a single elevated blood pressure measurement is not indicative of chronic hypertension in a patient. Due to avoidance of collecting data with identifiers, the authors were unable to longitudinally follow patients. The author’s proposal that higher rates of insurance lead to better overall health may also be complicated by self-selection bias (for example, persons who are covered by health insurance may be inherently more concerned about their health). Lastly, the data collection method was not done through random sampling. Persons who are more concerned about their health are also more likely to attend local community health fairs. It is unknown how this may affect the results.

## Conclusions

With the debate on health care currently consuming the politics of the United States, it is important to try and elucidate the real effect of health insurance on the well-being of the population. This study adds to the growing body of evidence suggesting that health insurance is a necessity in maintaining the health of the general population.
